# Zinc is an Antioxidant and Anti-Inflammatory Agent: Its Role in Human Health

**DOI:** 10.3389/fnut.2014.00014

**Published:** 2014-09-01

**Authors:** Ananda S. Prasad

**Affiliations:** ^1^Department of Oncology and Barbara Ann Karmanos Cancer Center, Wayne State University School of Medicine, Detroit, MI, USA

**Keywords:** zinc, anti-inflammatory agent, antioxidant agent, oxidative stress, inflammatory cytokines

## Abstract

Zinc supplementation trials in the elderly showed that the incidence of infections was decreased by approximately 66% in the zinc group. Zinc supplementation also decreased oxidative stress biomarkers and decreased inflammatory cytokines in the elderly. In our studies in the experimental model of zinc deficiency in humans, we showed that zinc deficiency *per se* increased the generation of IL-1β and its mRNA in human mononuclear cells following LPS stimulation. Zinc supplementation upregulated A20, a zinc transcription factor, which inhibited the activation of NF-κB, resulting in decreased generation of inflammatory cytokines. Oxidative stress and chronic inflammation are important contributing factors for several chronic diseases attributed to aging, such as atherosclerosis and related cardiac disorders, cancer, neurodegeneration, immunologic disorders and the aging process itself. Zinc is very effective in decreasing reactive oxygen species (ROS). In this review, the mechanism of zinc actions on oxidative stress and generation of inflammatory cytokines and its impact on health in humans will be presented.

## Introduction

Essentiality of zinc for human health was recognized only 50 years ago (1, 2). Currently, World Health Organization (WHO) estimates that nearly two billion subjects in the developing world may be zinc deficient. The clinical manifestations of zinc deficiency include growth retardation, testicular hypofunction, immune dysfunctions, increased oxidative stress, and increased generation of inflammatory cytokines (3–8). If severe zinc deficiency, as seen in patients with acrodermatitis enteropathica (AE), is not recognized and treated promptly with zinc administration, it may become fatal (9).

Zinc has now been used to treat and prevent diarrhea in infants and children throughout the world resulting in saving millions of lives (10, 11). Zinc is an effective therapeutic agent for the treatment of Wilson’s disease (12).

The severity and duration of common cold may be decreased significantly with the proper use of zinc lozenges (13, 14). The progression of age-related macular degeneration (AMD) and its complications and blindness in the elderly has been shown to be effectively managed by the use of the therapeutic zinc supplementation (15–18).

The studies of age-related eye diseases study group (AREDS) in AMD subjects has shown that during 10 years of follow-up, the mortality due to cardiovascular events in the elderly was significantly decreased in the zinc group (18). These clinical effects of zinc supplementation in humans are very impressive and have a high impact on human health.

The study of the roles of zinc in basic biochemical and molecular biology fields has also advanced tremendously during the past half a century. We now know that over 300 enzymes require zinc for their activation or stability of structures and there are over 2000 transcription factors that are involved in gene expression of proteins that are zinc-dependent (7). We have now learned that zinc is a molecular signal for immune cells and that homeostasis of intracellular Zn++ levels are maintained by 14 ZIP (SLC 39A) and 10 ZNT (SLC 30A) transporters.

In this review article, I will briefly describe the history of discovery of zinc as an essential element for humans and discuss the clinical manifestation of zinc deficiency and the role of zinc in cell-mediated immunity and as an antioxidant and anti-inflammatory agent in humans.

## Discovery of Human Zinc Deficiency

**Figure 1:**
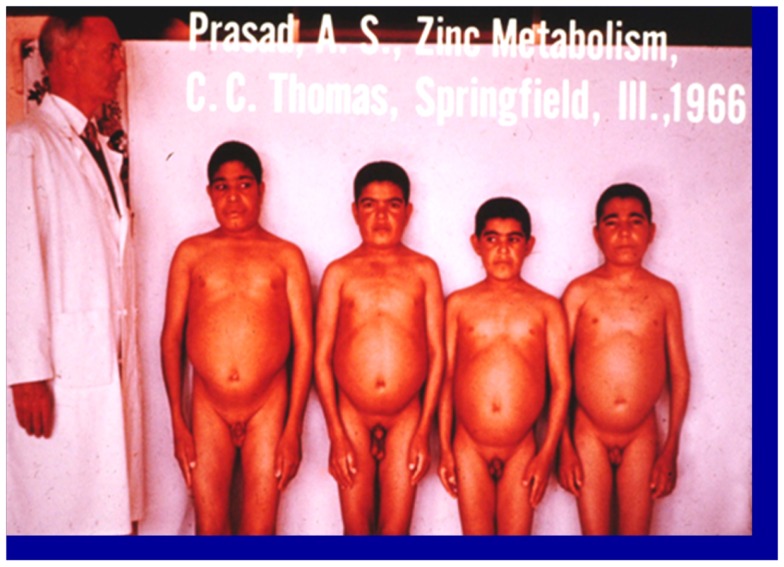
**A picture of four dwarfs from Iran**. From left to right (i) Age 21 years, height 4 ft, 11 (1/2″); (ii) age 18 years, height 4 ft 9″; (iii) age 18 years, height 4 ft 7″; (iv) age 21 years, height 4 ft 7″ Staff physician at left is 6 ft in height (1).

Zinc was known to be essential for the growth of plants and animals but its role in human health was not known until 1963 (2). The story of zinc in human health began when an Iranian physician presented to me a 21 years-old male patient who was severely anemic at the Medial Center grand round at Saadi Hospital, University of Shiraz Medical School. In the fall of 1958, following my training under Dr. C. J. Watson at the University of Minnesota Medical School as a clinical investigator, I went to Shiraz, Iran to teach the medical students in Shiraz Medical School. The patient was severely retarded in growth. Although he was 21-years-old chronologically, he looked like a 10-years-old male. He had infantile genitalia, rough and dry skin, mental lethargy, hepatosplenomegaly, and geophagia. He ate only bread made of whole wheat flour and the intake of animal protein was negligible. He also ate 1 pound of clay daily. This habit of clay eating (geophagia) was common in the villages around Shiraz, Iran (1). The anemia was severe. The hemoglobin was 5 g% and we determined that the patient was severely iron deficient. Although he was iron deficient, we found no evidence of blood loss (See Figure 1). Later, I discovered that this syndrome of iron deficiency, anemia, hepatosplenomegaly, hypogonadism, dwarfism, and geophagia was common in the villages near Shiraz, Iran (1). The extreme growth retardation affected approximately 10% of the villagers.

This interesting case presented two major clinical dilemmas for me. One was that how did this patient become severely iron deficient when there was no blood loss? The second problem was that I could not explain growth retardation and hypogonadism on the basis of iron deficiency, inasmuch as growth retardation and testicular atrophy are not observed in iron-deficient animals and humans. An examination of the Periodic Table suggested to me that deficiency of zinc may have been also present accounting for the growth failure and testicular hypofunction. We, therefore, hypothesized that a high phosphate content of their diet and geophagia may have caused malabsorption of both iron and zinc, resulting in the deficiency of both elements (1). In Shiraz, we had no facility to assay zinc in the plasma.

I subsequently moved to Cairo, Egypt and studied this syndrome extensively at the US Naval Medical Research Unit No. 3. My research was supported by the Vanderbilt University, School of Medicine, Department of Biochemistry and Medicine, and US Navy and NIH.

We observed that zinc concentrations in plasma, red blood cells, hair, and total zinc in 24-h urine were decreased in the Egyptian dwarfs. Zn^65^ studies showed that the plasma zinc turn-over rate was increased and the 24-h exchangeable pool was decreased in the dwarfs in comparison to the controls (2). We also showed that the zinc supplementation to these dwarfs resulted in 12.7–15.2 cm of longitudinal growth in 1 year and the genitalia size became normal within 3–6 months of zinc supplementation. Thus, our studies showed for the first time that zinc was essential for humans and that its deficiency occurred in the Middle East (3). The details of the circumstances leading to the discovery of human zinc deficiency have been recently published (19).

For nearly one decade, the suggestion that zinc deficiency occurred in humans remained very controversial. In 1974, however, National Research Council of the National Academy of Sciences declared zinc as an essential element for humans and established a recommended dietary allowance (RDA) (20). In 1978, FDA made it mandatory to include zinc in the total parenteral nutritional fluids (21).

It has been now estimated by WHO that nearly two billion subjects may be zinc deficient in the developing countries. This is due to the fact that most of these populations consume mainly bread made of whole wheat flour, which is high in phosphate compound that decreases the absorption of both iron and zinc. The phytate to zinc molar ratio >20 in a diet is unfavorable for zinc absorption and this may lead to zinc deficiency.

In the developed countries, zinc deficiency is also prevalent in the elderly population (approximately 30% may be zinc deficient). 25–30% of the Black-Americans and Mexican-Americans are also zinc deficient and approximately 25% of the pre-menopausal women of the child bearing ages are vulnerable to zinc deficiency (7, 22).

Conditioned deficiency of zinc in patient with alcoholic cirrhosis of the liver, chronic alcoholism with hyperzincuria, chronic renal disease, patients with malabsorption syndrome, and patients with sickle cell disease (SCD) who exhibit hyperzincuria due to persistent hemolysis are some of the examples of conditioned deficiency of zinc commonly seen in the world (7). Patients with chronic diseases including malignancies have poor appetite, and therefore, they are vulnerable to zinc deficiency. It is, thus, clear that zinc deficiency is fairly common in clinical practice but sadly the clinicians do not recognize this problem.

## Clinical Manifestations of Zinc Deficiency

There is a spectrum of clinical deficiency of zinc in humans. The symptoms may be very severe and even fatal if not recognized and treated promptly by zinc such as seen in patients with AE. AE is a genetic disorder mainly affecting infants of Italian, Armenian, or Iranian lineage (7, 9). The manifestations include bullous pustular dermatitis, particularly around the orifices. Opthalmic signs include blepharitis, conjunctivitis, photophobia, and corneal opacities. Neuropsychiatric signs include irritability, emotional instability, tremors and cerebellar ataxia, weight loss, growth failure, and male hypodonadism are prominent features. Congenital malformation of fetuses and infants born of pregnant women with AE has been reported frequently (23).

Acrodermatitis enteropathica patients are very susceptible to infections. Thymic hypolasia and plasmacytosis in the spleen are commonly seen in experiment animals. T cell-mediated immune disorders are corrected by zinc supplementation. Without zinc supplementation, the clinical course is downhill with failure to thrive and complicated by intercurrent bacterial, viral, fungal, and opportunistic infections. Gastrointestinal manifestations include diarrhea, malabsorption, steatorrhea, and lactose intolerance. Zinc supplementation in therapeutic doses (in excess of 50 mg elemental zinc daily) results in complete recovery.

Acrodermatitis enteropathica gene has been localized to a ~3.5 cm region on 8q 24 chromosome. The gene encodes for ZIP-4 known as one of the zinc transporter. In AE, mutations of this gene have been documented (24). Because of this mutation, intestinal absorption of zinc is affected adversely.

Severe deficiency of zinc has been observed in patients who receive total parenteral nutrition without zinc for prolonged period (25). Zinc is now routinely included in parenteral fluids to prevent this complication. Severe deficiency of zinc has also been observed in a patient with Wilson’s disease, who received penicillamine, which reduces the copper burden (26).

In moderate deficiency of zinc, the clinical manifestations include growth retardation, testicular failure, rough skin, poor appetite, mental lethargy, delayed wound healing, T cell-mediated immune dysfunction, and neurosensory disorders (7). Females are equally susceptible to zinc deficiency and their ovarian functions are also affected adversely due to zinc deficiency (7). Moderate deficiency of zinc is commonly seen in patients with nutritional deficiency of zinc throughout the world and many patients who develop conditioned deficiency of zinc.

The recognition of mild deficiency of zinc is difficult. In order to characterize this, we developed a human model of experimental mild zinc deficiency state in human volunteers. This was accomplished by the use of a semi-purified experimental diet, which supplied 3.0–5.0 mg of zinc daily (27). All other essential nutrients were adequate meeting RDA (RDA). The details of these experiments have been published earlier (27).

In this model, as a result of zinc deficiency, we observed decreased serum testosterone level, oligospermia, decreased NK cell (natural kill cell) lytic activity, decreased IL-2 activity of T helper cells, decreased serum thymulin activity (thymulin is a thymic hormone essential for the development and maturation of T cells), hyperammonemia, hypogeusia, decreased dark adaptation, and decreased lean body mass (27–29). It is clear from this study that even a mild deficiency of zinc in humans may affect clinical, biochemical, and immunological functions adversely.

Currently, the most widely used test for diagnosing zinc deficiency in humans is the determination of plasma zinc level. In our laboratory, normal levels of plasma zinc in adults and children are 100 ± 10 μg/dl. Values below 80 μg/dl will be considered to be in the deficient range. Measurement of plasma zinc by flameless atomic absorption specifically is useful provided the sample is not hemolyzed or contaminated. In patients with acute stress or infection, zinc from the plasma pool may redistribute to other compartments, thus making the diagnosis of zinc deficiency difficult. Intravascular hemolysis would also increase the plasma zinc level, inasmuch as the zinc in the red cells is much higher than in the plasma (7). Plasma zinc level of <50 μg/dl would be indicative of severe deficiency of zinc.

In our experience, determination of zinc in the lymphocytes, plasma somatomedin activity, and measurement of IL-2 mRNA in PHA stimulated mononuclear cells by reverse transcriptase (RT)-polymerase chain reaction (PCR) are the most sensitive tests for diagnosing zinc deficiency in humans (7). Zinc supplementation to humans corrects the plasma zinc levels and all other parameters mentioned above.

Recommended daily dietary allowances (RDA) for infants up to 1 year is 3–5 mg, for children from 1 to 10 years is 10 mg, and for adults (both males and females) it is 15 mg. For pregnant women, RDA is 20 mg and for lactating women RDA is 25 mg.

## Oxidative Stress and Chronic Inflammation in the Elderly

Oxidative stress is an important contributing factor for several chronic diseases attributed to aging, such as atherosclerosis and related cardiac disorders, cancer, neurodegeneration, immunologic disorders, and the aging process itself (5, 6). Together, ${\;}^{\cdot }{\text{O}}_{2}^{-}$, H_2_O_2_, and ^⋅^OH are known as reactive oxygen species (ROS) and they are continuously being produced *in vivo* under aerobic conditions. In eukaryotic cells, the mitochondrial respiratory chain, microsomal cytochrome P450 enzymes, flavoprotein oxidases, and peroxisomal fatty acid metabolism are the most significant intracellular sources of ROS (6). The nicotinamide adenine dinucleotide phosphate (NADPH) oxidases are a group of plasma membrane-associated enzymes, which catalyze the production of ${\;}^{\cdot }{\text{O}}_{2}^{-}$, from oxygen by using NADPH as the electron donor (6, 7). Zinc is an inhibitor of NADPH oxidase, which results in decreased generation of ROS. Zinc is also a co-factor of super oxide dismutase (SOD) an enzyme that catalyzes the dismutation of ${\;}^{\cdot }{\text{O}}_{2}^{-}$, to H_2_O_2_. Zinc also induces the generation of metallothionein, which is very rich in cysteine and is an excellent scavenger of ^⋅^OH.

Although the role of zinc as an antioxidant in cell cultures and animal models have been observed earlier, our studies showed for the first time the role of zinc as an antioxidant in the elderly.

We observed that zinc supplementation to healthy human subjects aged 20–50 years decreased the concentration of MDA (malondialdehyde), 4 hydroxy alkenals (HAE), and 8-hydroxy deoxyguanine in the plasma (30). This demonstrated that the oxidative stress markers were decreased by zinc supplementation in young adult human subjects (30). We also observed that the *ex vivo* induction of TNF-α and IL-1β mRNA in mononuclear cells were inhibited in zinc supplemented subjects and this resulted in decreased TNF-α induced nuclear factor-κB (NF-κB) activation in isolated MNCs (mononuclear cells) (30). We also reported that the gene expression of A20 and the binding of A20 transactivating factor to DNA were increased, resulting in the inhibition of NF-κB activation (30). NF-κB is involved in the gene expression of TNF-α and IL-1β in monocytes and macrophages in humans and HL-60 cells (human promyelocytic leukemia cell line, which differentiates to the monocyte and macrophage phenotype in response to PMA). This effect of zinc, inhibition of the gene expression of TNF-α and IL-1β in these cells is cell specific (30).

In order to understand the mechanism of zinc effect on cell-mediated immunity, we utilized RT-PCR analysis to determine PHA (phytohemagglutinins) induced expression of IL-2 mRNA in isolated MNCs in elderly subjects before and after zinc supplementation. Since zinc supplementation to younger subjects decreased the generation of inflammatory cytokines and decreased oxidative stress markers (30), we hypothesized that zinc supplementation would not only increase the generation of IL-2 in MNCs but also decrease the generation of inflammatory cytokines and decrease oxidative stress in the elderly.

We recruited 50 healthy elderly subjects of both sexes (aged 55–87 years) and all ethnic groups from a senior citizen center in Detroit, MI, USA to participate in a randomized, placebo-controlled trial of the efficacy of zinc with respect to the incidence of infections and the effect on *ex vivo* generated inflammatory cytokines and plasma oxidative stress markers (5).

Exclusion criteria included life expectancy of <8 months, progressive neoplastic disease, severe cardiac dysfunction, significant kidney, and liver disease. We also excluded those who were self-supplementing with zinc, who were not mentally competent, and who could not provide informed consent. The zinc supplemented group received 45 mg elemental zinc as gluconate daily.

A comparison of baseline data between the younger subjects and the elderly subjects is shown in Table 1. Plasma zinc was lower and the percentage of cells producing IL-1β and TNF-α and the generated levels of these cytokines were significantly higher in the elderly subjects. Generated IL-10 was also significantly higher in the elderly. This cytokine is known to produce a negative effect on IL-2 generation by Th1 cells. The plasma oxidative stress markers also were significantly higher in the elderly in comparison to the younger adults.

**Table 1:** **A comparison of selected variables in young adults (18–54 years old) and in older subjects (>55 years old)**.

Variables^a^	Young adults	Older subjects	*p* Value^c^
Plasma zinc (μg/dl)	101.4 ± 10.0^a^ (31)^b^	94.3 ± 11.4 (49)	0.046
Plasma ICAM-1 (ng/ml)	538 ± 112.7 (25)	652.6 ± 169.8 (47)	0.001
Plasma VCAM-1 (ng/ml)	1766 ± 480.4 (25)	2209 ± 890.5 (46)	0.008
Plasma E-selectin (ng/ml)	32.2 ± 13.1 (19)	6 ± 47.6 (69)	0.001
Plasma NO (μM)	42.7 ± 10.9 (24)	55.6 ± 14.7 (36)	0.001
Plasma MDA (μM)	0.36 ± 0.10 (16)	0.49 ± 0.15 (34)	<0.001
IL-1 β (% cells)	0.5 ± 9.2 (28)	0.4 ± 23.5 (48)	0.023
IL-1 β generated (pg/ml)	0.5 ± 110.9 (31)	0.3 ± 423.3 (28)	0.004
TNF-α (% cells)	10.18 ± 10.86 (22)	18.25 ± 20.5 (48)	0.035
TNF-α generated (pg/ml)	1522 ± 390 (26)	1882 ± 722.6 (24)	0.036

*Ref (5)*.

*^a^Values represent mean ± SD; ^b^number of subjects; ^c^*t*-test*.

Table 2 shows the effect of zinc supplementation on clinical variables. The mean incidence of infections in 12 months was significantly lower (*p* < 0.01) in the zinc supplemented group than in the placebo group. In the zinc supplemented group, the total incidence of infections in 12 months was 7, whereas in the placebo group it was 35.

**Table 2:** **Effect of zinc and placebo supplementation on clinical variables**.

Variables	Percentage of subjects affected in 1 year		
	Zinc group (*n* = 24)	Placebo group (*n* = 25)	Chi square Fishers exact test, *p*
Infection	29	88	<0.001
Upper respiratory
Tract infection	12	24	0.136
Tonsillitis	0	8	0.255
Common cold	16	40	0.067
Cold sores	0	12	0.124
Flu	0	12	0.124
Fever	0	20	0.027

One infection each/year	29%	52%	
Two infections each/year	0	24%	
Three infections each/year	0	8%	
Four infections each/year	0	4%	
Received antibiotics	8%	48%	

*Ref (5)*.

*Each person could appear in more than one sub-category of infection*.

The changes in plasma markers of oxidative stress (MDA + HAE and 8-OHdG) between baseline and at the end of 6 months of zinc supplementation showed a greater significant decrease compared to the placebo group (6) (Table 3).

**Table 3:** **Effect of zinc and placebo supplementation on plasma oxidative stress markers**.

	Baseline	6 months	*p* = (time × group^a^)
MDA + HAE (μmol/l)
Zinc suppl.^b^	1.66 ± 0.34^c^	1.35 ± 0.18	0.0002
Placebo suppl.^c^	1.70 ± 0.30	1.71 ± 0.35	
8-OHdG (ng/ml)
Zinc suppl.	0.63 ± 0.16	0.50 ± 0.14	0.030
Placebo suppl.	0.66 ± 0.13	0.68 ± 0.13	
Nitric oxide (μmol/l)
Zinc suppl.	87.34 ± 8.08	79.01 ± 10.96	0.180
Placebo suppl.	89.43 ± 11.72	86.74 ± 9.28	

*Ref (5)*.

*^a^*p* Value for change in groups over time. Multivariate repeated measures analyses were used to examine measures over time. ^b^Zinc (*n* = 13) or placebo (*n* = 11) supplemented subjects. ^c^Values represent mean ± SD. There were no significant differences (*t*-test) in oxidative stress markers between the two groups at baseline*.

With time (12 months of zinc supplementation), the plasma zinc in the zinc group increased significantly. Whereas in the placebo group, it tended to remain lower. Also with time, the *ex vivo* generation of TNF-α decreased significantly in the zinc group and increased significantly in the placebo group. The reduction in TNF-α concentration was maximal at the end of 6 months. *Ex vivo* generation of IL-10 decreased non-significantly in the zinc group. Tables 4 and 5 show the changes in the plasma zinc levels following supplementation.

**Table 4:** **Effect of zinc and placebo supplementation on interleukin (IL)2 mRNA and plasma zinc concentration in zinc-deficient elderly subjects**.

	Baseline	6 months	*p* = (time × group^a^)
IL-2 mRNA^b^
Zinc suppl.^c^	0.38 ± 0.07^d^	0.63 ± 0.03	<0.001
Placebo suppl.^d^	0.40 ± 0.05	0.39 ± 0.04	
Plasma Zn^e^
Zinc suppl.	84.0 ± 3.03	97.6 ± 5.98	<0.0088
Placebo suppl.	0.8 ± 2.04	89.2 ± 3.06	

*Ref (5)*.

*^a^*p* Value for change in groups over time. Multivariate repeated measures analyses were used to examine measures over time. ^b^Relative expression of IL-2 mRNA/18S. ^c^Zinc supplemented (*n* = 6) or placebo supplemented (*n* = 6) zinc-deficient elderly subjects. ^d^Values represent mean ± SD. There was no significant difference (*t*-test) in IL-2 mRNA between the two groups at baseline. In spite of the random assignment of zinc-deficient subjects into zinc and placebo group, the plasma zinc level was lower in the zinc group in comparison to the placebo group (*p* = 0.016). ^e^Plasma zinc levels (microgram per deciliter)*.

**Table 5:** **Changes in plasma zinc, oxidative stress makers, and inflammatory cytokines/molecules in zinc supplemented (Zn supp) elderly subjects^a^ (6)**.

Group	*n*	Pre	Post	*P*^b^	Change^c^	*ΔP*^d^
Zinc (μM)
Placebo	20	92.0 ± 3.8^e,f^	90.8 ± 5.0	0.134	−1.17 ± 4.59	<0.0001
Zn supp	20	91.9 ± 7.4^e^	101.5 ± 9.2	0.00006	9.52 ± 8.88	
MDA + HAE (μM)
Placebo	14	1.66 ± 0.37^e^	1.68 ± 0.35	0.357	0.019 ± 0.186	0.002
Zn supp	14	1.59 ± 0.40^e^	1.29 ± 0.26	0.0011	−0.30 ± 0.30	
Antioxidant power (U/mL)
Placebo	20	6.6 ± 2.2^e^	6.5 ± 1.7	0.417	−0.11 ± 2.32	0.0258
Zn supp	20	6.0 ± 1.6^e^	7.6 ± 1.9	0.0001	1.56 ± 2.23	
hsCRP (μg/L)
Placebo	20	2.14 ± 1.71^e^	2.49 ± 1.94	0.149	0.36 ± 1.45	0.0298
Zn supp	20	2.46 ± 1.91^e^	1.90 ± 1.51	0.015	−0.55 ± 1.05	
IL-6 (pg/mL)
Placebo	20	5.42 ± 3.47^e^	7.15 ± 4.56	0.026	1.74 ± 3.76	0.0031
Zn supp	20	8.34 ± 7.13^e^	5.44 ± 4.85	0.013	−2.94 ± 5.46	
MCP-1 (pg/mL)
Placebo	20	496.5 ± 154.0^e^	570.4 ± 205.4	0.011	74.1 ± 133.3	0.0113
Zn supp	20	531.5 ± 142.7^e^	506.8 ± 131.0	0.136	−24.25 ± 97.2	
sPLA (U/mL)
Placebo	20	76.0 ± 25.8^e^	100.6 ± 28.8	0.001	24.6 ± 30.9	0.006
Zn supp	20	73.3 ± 34.6^e^	70.0 ± 32.2	0.314	−3.23 ± 29.5	
sVCAM-1 (ng/mL)
Placebo	20	2102.9 ± 415.1^e^	2297.6 ± 358.2	0.0024	194.7 ± 273.1	<0.0001
Zn supp	20	2208.0 ± 345.6^e^	2035.0 ± 267.8	0.001	−171.5 ± 218.5	
sICAM-1 (ng/mL)
Placebo	20	321.1 ± 89.1^e^	302.7 ± 105.6	0.307	−18.40 ± 160.4	0.830
Zn supp	20	301.3 ± 68.9^e^	292.4 ± 75.6	0.365	−8.90 ± 113.6	
sE-selectin (ng/mL)
Placebo	20	54.7 ± 8.3^e^	57.5 ± 7.3	0.023	2.73 ± 5.70	0.0068
Zn supp	20	57.2 ± 9.7^e^	51.6 ± 8.6	0.023	−5.65 ± 11.7	

*^a^Pre, baseline; Post, after 6 months; MDA + HAE, malondialdehyde and hydroxyalkenals; hsCRP, high-sensitivity C-reactive protein; IL-6, interleukin-6; MCP-1, macrophage chemoattractant protein 1; sPLA, secretory phospholipase A2; sVCAM-1, soluble vascular cell adhesion molecule 1; sICAM-1, soluble intercellular adhesion molecule 1; sE-selectin, soluble E-selectin. ^b^Pre compared with Post values (paired *f*-test). ^c^Post minus Pre values. ^d^*P* for differences (Pre compared with Post) between placebo and zinc groups *(t-*test). ^e^There was no significance of plasma zinc and all biomarkers between placebo and Zn supp groups before supplementation (Pre). ^f^Mean ± SD (all such values)*.

In MNCs isolated from zinc-deficient elderly, zinc supplementation increased the *ex vivo* PHA-induced IL-2 mRNA and plasma zinc concentration, whereas placebo treated zinc-deficient subjects showed no such changes (5, 6) (Table 4). Our study in the elderly showed that zinc supplementation decreased the incidence of infections significantly (5). Zinc deficiency not only affects adversely the thymulin (a thymic hormone) activity but also decreases the generation of IL-2 and IFN-γ from Th1 cells. Zinc deficiency also decreases IL-12 generation from macrophages. IL-12 and IFN-γ are required for optimal phagocytic activity of macrophages against parasites, viruses, and bacteria.

**Figure 2:**
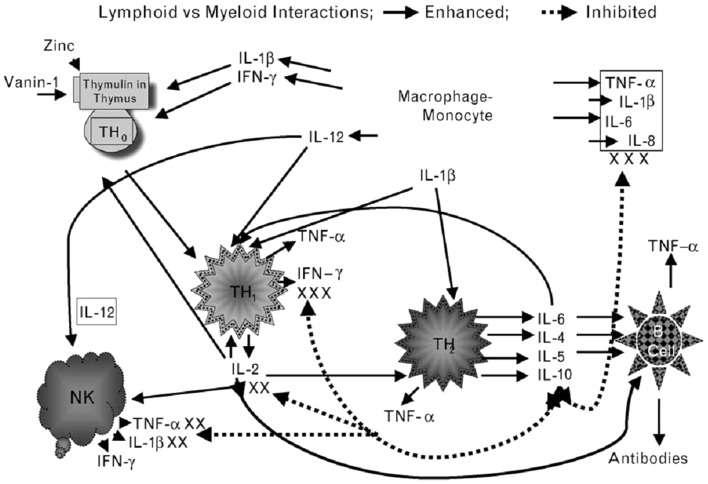
**Zinc is an integral part of a thymic hormone molecule, thymulin**. Thymulin is required for maturation of T cells. Zinc deficiency-induced decrease in thymulin activity is associated with decreased maturation of T cells and Th1 production of IL-2 and INF-γ. Decreased IL-2 leads to decreased NK and T cytolytic cell activities. Macrophages–monocytes produce IL-12 (a zinc-dependent cytokine), which along with INF-γ kills parasites, viruses, and bacteria. Th2 cytokines, in general are not affected by zinc deficiency except IL-10, which may be increased in zinc-deficient elderly individuals. Increased IL-10 from Th2 cells further affects Th1 functions adversely. Thus, in zinc deficiency there is a shift from Th1 to Th2 functions and cell-mediated immune functions are impaired. Zinc deficiency also leads to stress and activation of macrophages–monocytes, resulting in increased generation of inflammatory cytokines, IL-1β, IL-6, IL-8, and TNF-α. Solid lines indicate pathways leading to generation of selected cytokines and dotted lines represent pathways, which lead to inhibition of cytokine generation. NK represent natural-killer cells; Th1 represent activated Th1 type T cells and secreted cytokines (small triangles); Th2 represents activated Th2 type T cells and secreted cytokines (small rounds); B-cell represents B-cell lineages and associated immunoglobulins (triangles). (Prasad, AS. Zinc: role in immunity, oxidative stress and chronic inflammation. Current Opinion in Clin Nutr and Metab Care, 12:646–652, 2009)

In our experimental human zinc deficiency model, even a mild deficiency increased *ex vivo* generation of IL-1β by monocytes, suggesting that zinc deficiency *per se* may activate monocytes and macrophages to generate inflammatory cytokines and increase oxidative stress (28). Our study showed that zinc supplementation improved Th1 cells cytokines production, decreased generation of inflammatory cytokines, and decreased oxidative stress. Figure 2 summarizes our concept of the role of zinc in cell-mediated immunity.

## Effect of Zinc Supplementation on Oxidative Stress and Inflammatory Cytokines in the Elderly

Oxidative stress and inflammatory cytokines have been implicated in many chronic diseases in the elderly including atherosclerosis. It is a slowly progressive chronic inflammatory condition characterized by focal arterial lesions that ultimately occlude the blood vessels and may lead to angina, myocardial infarction, stroke, and sudden death. Classical risk factors for atherosclerosis include advanced age, smoking, hypertension, diabetes, and obesity. Recently chronic inflammation has been implicated in the development of atherosclerosis.

Nutritional deficiency of zinc is common not only in developing countries but also in certain groups of populations in the developed countries. We have reported that nearly 30% of the healthy elderly subjects may be zinc deficient (5, 6).

In healthy elderly zinc-deficient subjects, we showed increased concentrations of plasma lipid peroxidation by-products and endothelial cell adhesion molecules compared with those in younger adults (5, 6). We proposed that zinc may have an atheroprotective function because of its anti-inflammatory and antioxidant properties (6).

We supplemented 20 elderly subjects, ages >65 years with 45 mg elemental zinc as gluconate daily for 6 months. Twenty elderly subjects’ ages >65 years received placebo. This was a double blind placebo-controlled trial. In the zinc group, plasma zinc increased, plasma antioxidant power increased, and plasma oxidative stress marker decreased in comparison to the placebo group (6).

Zinc supplementation resulted in significant decreases in hsCRP, plasma IL-6 concentration, MCP-1, sPLA (secretory phospholipase A), SE-selectin (soluble E-selection), and sVCAM-1 after 6 months of supplementation compared with the placebo group (6). Our analysis also showed that the plasma zinc concentrations were inversely correlated with the changes in concentrations of plasma hsCRP, MCP-1, MDA + HAE, and VCAM-1 after 6 months of zinc supplementation (Tables 5 and 6).

**Table 6:** **Effect of zinc on tumor necrosis factor-α (TNF-α), interleukin-1β (IL-1β), vascular cell adhesion molecule 1 (VCAM-1), and malondialdehyde and hydroxyalkenals (MDA + HAE) in HL-60 and THP-1 cells and human aortic endothelial cells (HAECs)^a^ (6)**.

	No stimulation			ox-LDL stimulation		
	Zn−	Zn+	*P*^b^	Zn−	Zn+	*P*^b^
HL-60 cells
TNF-α (pg/mL)	26.5 ± 25.4	12.1 ± 12.1	0.21	317.2 ± 119.7	152.7 ± 96.4	0.007
IL-1β (pg/mL)	1.4 ± 1.2	0.8 ± 0.7	0.52	3.9 ± 1.4	1.3 ± 0.5	0.042
VCAM-1 (pg/mL)	18.3 ± 4.7	14.2 ± 1.7	0.073	69.9 ± 2.8	32.1 ± 4.3	0.006
MDA + HAE (μM) THP-1 cells	2.6 ± 0.7	1.5 ± 0.6	0.001	5.6 ± 1.4	2.3 ± 0.5	0.046
THP-1 cells
TNF-α (pg/mL)	32.2 ± 15.1	23.8 ± 12.0	0.022	181.2 ± 13.9	121.0 ± 17.9	0.027
IL-β (pg/mL)	1.5 ± 0.1	0.9 ± 0.4	0.027	4.4 ± 0.7	1.7 ± 0.9	0.004
MDA + HAE (μM)	1.4 ± 0.6	1.0 ± 0.5	0.013	4.5 ± 0.6	2.0 ± 0.7	0.004
HAECs
TNF-α (pg/mL)	8.0 ± 6.6	4.2 ± 5.0	0.06	22.6 ± 2.3	13.6 ± 2.1	0.034
IL-lβ (pg/mL)	5.8 ± 5.7	2.8 ± 2.7	0.11	13.1 ± 4.8	6.2 ± 1.8	0.028
VCAM-1 (ng/mL)	3.5 ± 1.5	4.5 ± 2.1	0.247	23.8 ± 4.7	13.8 ± 2.1	0.016
MDA + HAE (μm)	1.16 ± 0.36	1.02 ± 0.20	0.18	3.33 ± 1.02	1.45 ± 0.77	0.028

*^a^All values are means ± SDs. Zn−, zinc deficient; Zn+, zinc sufficient*.

*^b^For differences between Zn− (1 μM Zn) and Zn+ (15 μM. Zn) cell groups (Student’s *t-*test; *n* = 3)*.

C-reactive protein is widely used as a biomarker of chronic inflammation and prognosis in patients with cardiovascular disease (6). Ours is the first observation that zinc supplementation downregulates hsCRP in humans.

The increased generation of ROS and the activation of redox-dependent signaling cascades are involved in atherosclerosis (6). ROS itself can upregulate NF-κB-mediated transcriptional activation of inflammatory genes (6), thereby potentially acting as independent triggers of atherosclerosis. Zinc supplementation decreased oxidative stress in cell culture models, animal models, and humans (6). Thus decreased oxidative stress by zinc in the elderly may decrease LDL oxidation and exhibit atheroprotecitve effect. Results of our studies in the elderly support this hypothesis.

## Zinc and Age-Related Macular Degeneration

Age-related macular degeneration affects nearly 25% of individuals older than 65 years of age, and late-stage disease accounts for nearly 50% of legal blindness in Europe and North-America (15–18). Newsome et al. (15) reported that zinc levels are decreased n human eyes with signs of AMD and suggested that zinc deficiency may lead to oxidative stress and retinal damage.

Age-related eye diseases study group supported by NIH, conducted an 11-center double blind clinical trial in patients with dry-type AMD (16, 17). Three thousand six hundred forty participants of age 55–80 years were enrolled for trial and their follow-up period was 6.3 years. Participants were randomly assigned to receive daily orally one of the following: (1) antioxidants (Vitamin C 500 mg, Vitamin E 400 IU, and β-Carotene 15 mg; (2) zinc 80 mg as zinc oxide and copper 2 mg as copper oxide to prevent copper deficiency induced by therapeutic level of zinc; (3) antioxidants plus zinc, or (4) placebo.

In the group taking zinc supplements, advanced AMD was decreased by ~21% and loss of vision was prevented in 11%. In the group taking the vitamins alone, advanced AMD was decreased by 17% and vision loss was decreased by 10%. In the group taking both zinc and the vitamins, the advanced AMD decreased by 25% and the vision loss was decreased by 19% (16, 17). No significant side effects were noted in subjects who received therapeutic levels of zinc (16, 17). In the group taking both zinc and the vitamins, the advanced AMD decreased by 25% and the vision loss was decreased by 19% (16, 17). Interestingly, only the zinc supplemented group showed increased longevity (17). The risk of mortality was reduced by 27% in the AREDs studies in subjects who received only therapeutic levels of zinc daily. Most importantly, a later follow-up study showed that the reduction in mortality was due to a decrease in death caused by cardiovascular diseases, suggesting a beneficial effect of zinc on atherosclerosis (18).

## Mechanism of Zinc Action as Anti-Inflammatory Agent: Studies in Cell Culture Models

We studied the effect of zinc on inflammatory cytokines and oxidative stress markers in HL-60 (human promyelocytic leukemia cell line), THP-1 (human monocytic leukemia cell line), and HAEC (human aortic endothelial cells) (6, 30). The generation of TNF-α (tumor necrosis factor-α), IL-1β, VCAM-1, and MDA + HAE in HL-60, THP-1 cells, and HAECs after incubation with ox-LDL for 24 h were significantly decreased in zinc sufficient cells in comparison to the zinc-deficient cells. Zinc increased A20 and PPAR-α generation in ox-LDL stimulated THP-1 and HAEC cells compared with zinc-deficient cells. After 24 h of ox-LDL stimulation, zinc sufficient THP-1 and HAEC cells showed a significant decrease in NF-κB activation compared to zinc-deficient cells (6).

NF-κB is one of the major immune response transcription factors involved in molecular signaling. Zinc plays an important role in activation of NF-κB. The regulation of NF-κB activation by zinc is, however, cell specific (30). We have reported earlier that zinc is required for NF-κB DNA binding in purified or recombinant NF-κB p50 protein in T helper cell lines (31). We reported that normal healthy volunteers who were supplemented with 45 mg elemental zinc daily had a significant decrease in TNF-α and IL-1β messenger RNAs and TNF-α induced NF-κB DNA binding in isolated peripheral blood mononuclear cells in comparison to placebo treated subjects (30). Additionally, zinc upregulated the expression of A20 in HL-60 cells. Our study showed that zinc decreased ox-LDL-induced generation of TNF-α, IL-1β, and VCAM-1, oxidative stress markers in the plasma and activation of NF-κB, and increased A20 and PPAR-α protein in human monocytic and vascular endothelial cells (6). We proposed that zinc inhibited NF-κB activation via A20, a zinc finger transactivating factor that plays an important role in down regulating IL-1β and TNF-α induced NF-κB activation (6). A20 was originally thought to protect cells from TNF-α-induced cytotoxicity by inhibiting the activation of NF-κB resulting in decreased IL-1β and TNF-α signaling in endothelial cells (6). It was reported that A20 inhibits NF-κB signaling by TNF-α and IL-1β via TNF-receptor-associated factor pathways in endothelial cells (6).

The PPAR-α and -γ of nuclear receptors, the mediators of lipoprotein metabolism, inflammation, and glucose homeostasis were shown to play a protective role in the development and progression of atherosclerosis. The mechanism by which zinc may exhibit atheroprotective role is most likely due to its anti-inflammatory effect. We showed that zinc-sufficient HAEC cells had an increase in PPAR-α concentration compared with zinc-deficient HAEC cells, which suggested that zinc increases the expression of PPAR-α protein, which may contribute to down-regulation of inflammatory cytokines and adhesion molecules. We conclude that down-regulation of NF-κB activation by zinc via A20-PPAR-α signaling pathways results in decreased generation of inflammatory cytokines, which protects the endothelial cells from atherosclerosis.

## Proposed Concept of Mechanism of Zinc Action as an Antioxidant and Anti-Inflammatory Agent

**Figure 3:**
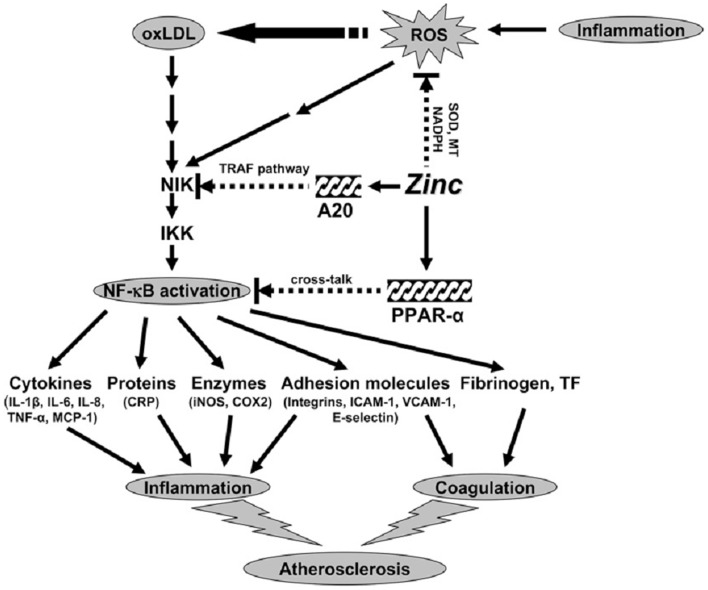
**Signaling pathway for zinc prevention of atherosclerosis in monocytes/macrophages and vascular endothelial cells: a proposed hypothesis**. Reactive oxygen species (ROS) induced by many stimuli modifies LDL into oxidized LDL (ox-LDL) in macrophages and vascular endothelial cells. ox-LDL or ROS can activate the apoptotic pathway via activation of proapoptotic enzymes and the nuclear transcription factor κB (NF-κB) pathway via NF-κB inducible kinase (NIK) activation, which eventually results in the development and progression of atherosclerosis. Zinc might have an atheroprotective function by the following mechanisms: (1) inhibition of ROS generation via metallothionein (MT), superoxide dismutase (SOD), and NADPH, and (2) down-regulation of atherosclerotic cytokines/molecules such as inflammatory cytokines, adhesion molecules, inducible nitric oxide synthase (iNOS), cyclooxygenase 2 (COX2), fibrinogen, and tissue factor (TF) through inhibition of NF-κB activation by A20-mediating tumor necrosis factor (TNF)-receptor associated factor (TRAF) signaling and peroxisome proliferator-activated receptor a (PPAR-a)-mediating crosstalk signaling. The black arrows indicate up-regulation; arrows with a broken line indicate down-regulation or the inhibitory pathway. IKK, I-κB kinase; IL, interleukin; MCP-1, macrophage chemoattractant protein 1; CRP, C-reactive protein; ICAM-1, intercell adhesion molecule 1; VCAM-1, vascular cell adhesion molecule 1 (14).

Our concept of the mechanism of zinc action as an antioxidant and anti-inflammatory agent is shown in Figure 3. Inflammation generates oxidative stress by increasing ROS, which results in oxidation of LDL. Oxidized LDL activates the NF-κB inducible kinase/IK-β kinase/NF-κB signaling pathway and upregulates its downstream target genes such as inflammatory cytokines, CRP, adhesion molecules, inducible nitric oxide synthase, cyclooxygenase 2, fibrinogen, and tissue factor. These cytokines and molecules attract neutrophils, monocytes, macrophages, and platelets, induce coagulation, and initiate development of atherosclerosis. Our study showed that zinc supplementation increased plasma antioxidant power, decreased plasma inflammatory cytokines, and oxidative stress biomarkers in the elderly subjects.

Zinc decreased NF-κB activation and its target genes such as TNF-α, IL-1β, and VCAM and increased the gene expression of A20 and PPAR-α, the two zinc finger proteins with anti-inflammatory properties in HL-60 and THP-1 cells and HAECs after ox-LDL stimulation. Thus, zinc decreased the expression of these cytokines and molecules by inhibition of NF-κB activation via A20 and PPAR-α pathways.

## Mechanism of Zinc Action as an Antioxidant

Zinc functions as an antioxidant by different mechanisms. Firstly, zinc competes with iron (Fe) and copper (cu) ions for binding to cell membranes and proteins, displacing these redox active metals, which catalyze the production of ^⋅^OH from H_2_O_2_. Secondly, zinc binds to (SH) sulfhydryl groups of bio-molecules protecting them from oxidation. Thirdly, zinc increases the activation of antioxidant proteins, molecules, and enzymes such as glutathione (GSH), catalase, and SOD and also reduces the activities of oxidant-promoting enzymes such as inducible nitric acid synthase (iNOS) and NADPH oxidase and inhibits the generation of lipid peroxidation products (32). Fourthly, zinc induces the expression of a metal-binding protein metallothionein (MT), which is very rich in cysteine and is an excellent excavanger of ^⋅^OH ions (32).

Nuclear factor erythroid 2-related factor 2 (Nrf2), a family member of cap’n’ collas/basic leucine zipper (CNC-bZIP) proteins is a critical transcription factor that regulates the gene expression of antioxidant proteins and enzymes such as GSH and SOD, as well as detoxifying enzymes such as glutathione-*S*-transferase-1 (GSTA1) and hemeoxygenase-1 (HO-1), by binding to an antioxidant responsive element (ARE) in the promoter region of the target gene (32). Several studies have shown that zinc may have a regulatory role in Nrf2. Zinc upregulates Nrf2 activity and decreases oxidative stress (32).

## Conclusion

Deficiency of zinc in humans was first reported nearly 50 years ago. The current estimate of the WHO is that nearly two billion subjects worldwide may have nutritional deficiency of zinc. This is because populations subsisting on high cereal protein diets have high intake of phytate, an organic phosphate compound, which complexes zinc and makes it unavailable for absorption. Major effects of zinc deficiency are growth retardation, hypogonadism, cell-mediated immune dysfunctions, increased oxidative stress, and increased generation of inflammatory cytokines. Zinc is a molecular signal for immune cells. Zinc is required for differentiation and generation of T helper cells. Generation of mRNA s of IL-2 and IFN-γ by Th1 cells are zinc-dependent and zinc-dependent transcription factors are involved in this process. Zinc supplementation to elderly subjects resulted in decreased incidences of infections, decreased plasma oxidative stress markers, and decreased generation of inflammatory cytokines and increased plasma zinc levels. Inasmuch as chronic inflammation and oxidative stress are implicated in many chronic diseases of the elderly, we hypothesize that zinc supplementation to the elderly may be very beneficial.

## Conflict of Interest Statement

The author declares that the research was conducted in the absence of any commercial or financial relationships that could be construed as a potential conflict of interest.
